# Good glycemic control of gestational diabetes mellitus is associated with the attenuation of future maternal cardiovascular risk: a retrospective cohort study

**DOI:** 10.1186/s12933-019-0881-6

**Published:** 2019-06-05

**Authors:** Enav Yefet, Naama Schwartz, Basma Sliman, Avraham Ishay, Zohar Nachum

**Affiliations:** 1 Department of Obstetrics & Gynecology, Emek Medical Center, Afula, Israel; 2 Research Authority, Emek Medical Center, Afula, Israel; 3 Endocrine & Diabetes Unit, Emek Medical Center, Afula, Israel; 4 Rappaport Faculty of Medicine, Technion, Haifa, Israel

**Keywords:** Gestational diabetes mellitus, Pregnancy, Glycemic control, Type 2 diabetes mellitus, Hypertension, Obesity, Dyslipidemia, Metabolic syndrome

## Abstract

**Background:**

To examine whether glycemic control of gestational diabetes mellitus (GDM) could modify the risk for future maternal metabolic and cardiovascular morbidities.

**Methods:**

A retrospective cohort study of women with a first diagnosis of GDM who delivered between 1991 and 2011. Women were divided into groups of good and poor glycemic control, defined as a mean daily glucose of up to 95 mg/dL (N = 230) and more than 95 mg/dL (N = 216), respectively. In addition, a control group of women without GDM (N = 352) was also analyzed. The primary outcomes were the development of type 2 diabetes mellitus (T2DM), obesity, hypertension, or dyslipidemia.

**Results:**

Mean follow-up time was 15.8 ± 5.1 years. Assessment was performed at a maternal age of 45 ± 7 years. The rates of the study outcomes in the control, GDM with good glycemic control and GDM with poor glycemic control were as follows: T2DM [19 (5.4%), 87 (38%), 127 (57%)]; hypertension [44 (13%), 42 (18%), 44 (20%)]; obesity [111 (32%), 112 (48%), 129 (58%)]; and dyslipidemia [49 (14%), 67 (29%), 106 (48%)]. Glycemic control was an independent risk factor for T2DM in multivariate Cox regression analysis (hazard ratio (HR) for poor glycemic control vs. controls 10.7 95% CI [6.0–19.0], good glycemic control vs. control HR 6.0 [3.3–10.8], and poor glycemic control vs. good glycemic control HR 1.8 [1.3–2.4]). Glycemic control was also an independent risk factor for dyslipidemia (poor glycemic control vs. controls HR 3.7 [2.3–5.8], good glycemic control vs. controls HR 2.0 [1.2–3.2], and poor glycemic control vs. good glycemic control HR 1.8 1.8 [1.3–2.6]). The fasting glucose level during oral glucose tolerance test (OGTT) was also an independent risk factor for these complications. The interaction term between glycemic control and the fasting value of the OGTT was not statistically significant, suggesting that the effect of glycemic control on the rate of future T2DM and dyslipidemia was not modified by the baseline severity of GDM.

**Conclusion:**

GDM and especially poor glycemic control are associated with T2DM and dyslipidemia. Strict glycemic control for reducing that risk should be evaluated in prospective trials.

## Background

Poor glycemic control during pregnancy with gestational diabetes mellitus (GDM) is a well-known cause of short-term maternal and neonatal complications such as an increased risk for spontaneous preterm birth [1], neonatal hyperbilirubinemia and hypoglycemia [2], cesarean sections, macrosomia, metabolic complications, shoulder dystocia, stillbirth, days in the neonatal intensive care unit, and respiratory complications [3]. Strict glycemic targets and early screening and management for GDM were shown to decrease the incidence of diabetes-related complications [2–4]. GDM was also shown to be associated with an increased risk for long-term maternal complications such as type 2 diabetes mellitus [5] and cardiovascular disease [6], as well as components of e metabolic syndrome including central obesity, hypertriglyceridemia, low HDL levels, hyperglycemia, and hypertension [7–9]. Glycemic control was shown to modify the risk for GDM recurrence in a subsequent pregnancy [10]. However, the effect of glycemic control during pregnancy on other long-term maternal metabolic complication has not been elucidated. It is not known whether GDM is a marker for future complications since they share a common pathogenesis or whether GDM is an independent risk factor for metabolic complications. If the latter hypothesis is true, good glycemic control may have a protective effect against future complications. Another issue of interest is examining the extent to which each complication is affected by a history of GDM, since the data in the literature is inconclusive; this, in large part, is due to heterogeneity in study designs and such confounders as ethnicity and body mass index (BMI) [11, 12].

The present study aimed to explore the association between GDM and cardio-metabolic morbidities and, more specifically, to examine whether good glycemic control is associated with a reduced risk of these complications.

## Methods

### Study design

A retrospective cohort study was conducted at the Gestational Diabetes Clinic and at the Obstetrics and Gynecology Department at Emek Medical Center, a university teaching hospital in Afula, Israel. This study was authorized by the local review board at Emek Medical Center (approval no. EMC-90-11) with an informed consent waiver due to its retrospective design.

The study population consisted of women with a first GDM diagnosis who delivered at Emek Medical Center between 1991 and 2011, and who had completed at least one consecutive birth at the same medical center. We included women with a second delivery at our institution because it increased the possibility of obtaining information on their current health status compared to women about whom we had no knowledge regarding their following obstetric follow-up. The GDM diagnostic criteria in our medical center remained the same throughout the entire study period. GDM diagnosis was established if the 50 g glucose challenge test (GCT) was ≥ 200 mg/dL, or if the 100 g oral glucose tolerance test (OGTT) had at least two abnormal values according to the Carpenter and Coustan criteria [13], or one abnormal value according to the 1979 National Diabetes Data Group (NDDG) [14]. This protocol accords with the regular departmental protocol. We used both diagnostic criteria since they are validated for GDM diagnosis and accepted by the American College of Obstetricians and Gynecologists (ACOG) and since choosing only one method might miss women with GDM.

The control group was randomly sampled from deliveries that took place during the GDM group study period and according to the sample size calculation. It included women without GDM among all available pregnancies in the medical center database during the study period. An index pregnancy (with either normal GCT or normal OGTT [84 women who did not complete a GCT or OGTT during the index pregnancy were excluded]) was chosen randomly to be included in the reference group.

We excluded women who did not complete a GCT or OGTT. Women without information regarding their current health status in the electronic database were also excluded (around 5% of the women).

Emek Medical Center serves a population of approximately 500,000 people residing in the cities, towns, and villages of northeast Israel. The National Health Insurance Law [15] provides that Israeli residents are entitled to equality of health services (quality and quantity).

The management of patients with GDM has been carried out at the Center’s Gestational Diabetes Clinic for the past 25 years. Women with GDM are referred to the clinic, where they are closely monitored by specialist physicians in order to achieve appropriate glycemic control. The routine follow up was as follows: the initial visit at the Gestational Diabetes Clinic included the recording of a full medical history by the clinic’s attending physician. In addition, each patient was instructed by a certified clinical nutritionist with regard to dietary and lifestyle recommendations for patients with diabetes. All women prescribed a diet ranging from 25 kcal/kg for overweight and obese women to 35 kcal/kg for women of normal weight that was divided into 3 full meals and 4 snacks consisting of 50% carbohydrates, 30% fat, and 20% protein. Glycemic control during pregnancy was evaluated by a daily chart that included 7 measurements: 3 pre-prandial, 3 post-prandial, and a 7th measurement at 10 p.m. The post-prandial measurements were taken 120 min after meals. The glucose chart was filled daily for a week, after which insulin was initiated if repeated pre-prandial glucose values were > 95 mg/dL, or repeated post-prandial values were > 120 mg/dL. Repeated elevated values were noted when at least 20% of the glucose measurements were elevated beyond the values described above. The daily glucose charts were continued until delivery and the same values were used to adjust insulin dosage.

### Establishing good and poor glycemic control groups

**Fig. 1:**
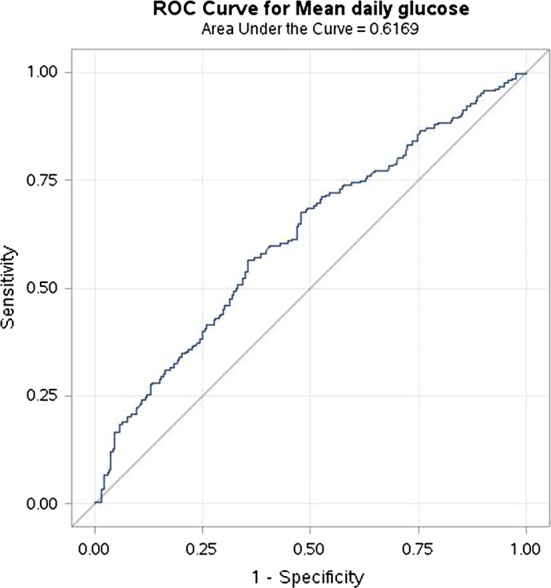
The ROC curve for the predictive probability of type 2 diabetes mellitus by mean daily glucose value according to the daily glucose charts of women with gestational diabetes mellitus. The ROC curve was statistically significant (AUC with 95% CI 62% [56–67%], *p* < 0.0001). *AUC* area under the curve, *CI* confidence interval, *ROC* receiver operating characteristic

Poor glycemic control was formerly determined according to the association between glycemic control and short-term GDM outcomes [3]. Since this study focused on long-term outcomes, we first wanted to establish the appropriate cutoff point for long-term complications. To this end, we performed a preliminary study in which we used all the available data regarding mean daily glucose which we could obtain from the GDM cohort’s daily glucose charts. We examined who developed type 2 diabetes mellitus later in life and performed an analysis of the predictive probability of mean daily glucose for this outcome using the receiver operating characteristic (ROC) curve (Fig. 1). We found that mean daily glucose was a statistically significant predictor of type 2 diabetes mellitus (area under the curve (AUC) 62%, 95% confidence interval [56–67%]). A cutoff of a mean daily glucose value of 95 mg/dL predicted the risk for type 2 diabetes mellitus with 57% sensitivity and 62% specificity. Thus, we considered good and poor glycemic control to be a mean daily glucose of up to 95 mg/dL and more than 95 mg/dL, respectively. Those groups were compared to each other and to the control group of women without GDM. It should be noted 95 mg/dL was also suggested in our previous study as increasing the risk for GDM recurrence, which is also a long-term outcome [10].

### Data collection

All the information including demographic and obstetrics data was obtained from the women’s medical records, laboratory systems, gestational diabetes clinic files, and delivery records. Data regarding long-term outcomes was extracted from our medical center’s electronic databases, which are also connected to community medical records. Those databases include information on patients’ diagnoses according to the ICD9, laboratory tests, and prescribed medications. The computerized system also issues an alert whenever an abnormal laboratory result is obtained. HbA1c results during the index pregnancy were not available for approximately half of the women and we therefore chose not to analyze this variable.

### Study outcome

The study’s primary outcomes were the development of type 2 diabetes mellitus, obesity, hypertension, or dyslipidemia (defined as pure or mixed hypercholesterolemia/hypertriglyceridemia).

A secondary outcome was the development of ischemic heart disease. Those outcomes were established primarily according to the patients’ diagnoses, which accords with ICD9 criteria. Information regarding laboratory tests and prescribed medications was also collected and assisted to confirm the diagnosis.

### Statistical analyses

The prevalence of hypertriglyceridemia, hyperglycemia, hypertension, and obesity in women aged 40–49 years was reported to be 23.7%, 30%, 24.5%, and 62%, respectively in a survey conducted in the USA [16]. We hypothesized that the risk for women without GDM or with GDM with good glycemic control would be 7% lower, and the risk for women with GDM with poor glycemic control would be 7% higher than the reported prevalence.

A sample size of 224 women for each group is sufficient for finding the study outcomes with 5% 2-sided alpha and at least 80% power as calculated by the Chi square test.

Categorical variables were analyzed using the Chi square test or Fisher’s exact test. The difference between the two groups’ continuous data was assessed using the t-test or Mann–Whitney U test when the data was not normally distributed.

We evaluated the risk of developing study outcomes over time by using the Kaplan–Meier curve from the time of the index pregnancy to the development of study outcomes as measured in years. A log-rank test was performed in order to compare the groups’ survival curves (*p* < 0.05 was considered significant). Confounders were explored using a stepwise multiple Cox regression which we used to assess independent risk factors for the study outcomes. If any of the study outcomes pre-existed prior to the index pregnancy or if the exact date of the diagnosis was unknown then those cases were removed from the Cox regression analysis of the specific outcome. Multivariate Cox regression was also utilized to examine the interaction between glycemic control and OGTT values with respect to the risk for the study outcomes. Finally, the components of the daily glucose charts, i.e. the mean pre-prandial and post-prandial glucose values were assessed as predictors for metabolic and cardiovascular morbidities.

Statistical analyses were carried out using the SAS statistical analysis software, version 9.4. Significance was set at *p* < 0.05.

## Results

**Fig. 2:**
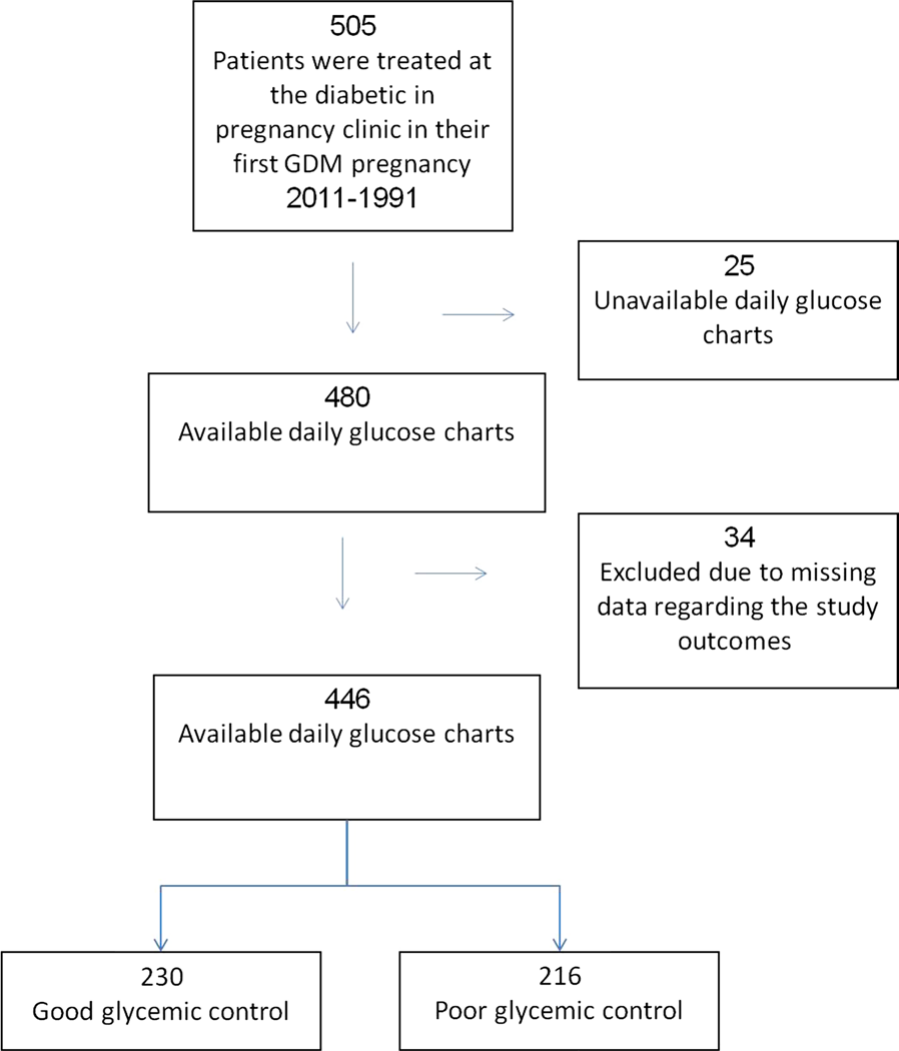
Patients’ flow chart

Mean follow up time for women with GDM and controls was 15.8 ± 5.1 and 15.7 ± 5.0 years, respectively (p = 0.8). Mean maternal age during assessment was 45 ± 7 years. Figure 2 presents the patients’ flow chart. Two hundred and thirty and 216 women had good and poor glycemic control during the GDM pregnancy, respectively. In 55 (24%) and 154 (71%) women insulin was used for glycemic control in the good and poor glycemic control groups, respectively. Of 446 women with GDM, 83 (19%) women developed type 2 diabetes mellitus before their subsequent pregnancy. Among the remaining 363 women, 203 (56%) developed GDM in their subsequent pregnancy. Demographic and obstetric characteristics of the index pregnancy are presented in Table 1. Women with good glycemic control had more cases of primiparity, and presented lower fasting and 3-h post-OGTT values compared to the poor glycemic control group.

**Table 1:** Demographic and obstetric characteristics of the study population and study outcomes

	Control N = 352	Good glycemic control N = 230	*p* value*	Poor glycemic control N = 216	*p* value^†^	*p* value^‡^
Age	28.2 (5.2) [27.9, 24.0–32.3]	29.7 (4.8) [29.1, 26.3–33.1]	0.0005	30.4 (4.9) [30.5, 26.9–34.0]	< 0.0001	0.12
Age ≥ 35	41 (12%)	31 (13%)	0.51	42 (19%)	0.01	0.09
Number of previous pregnancies	3.1 (2.1) [1–4]	2.8 (2.1) [1–4]	0.02	3.3 (2.3) [1–5]	0.41	0.006
First pregnancy	88 (25%)	80 (35%)	0.01	58 (27%)	0.64	0.07
Number of previous births	2.6 (1.7) [1–3]	2.3 (1.8) [1–3]	0.0002	2.7 (2.0) [1–4]	0.52	0.01
Primiparity	106 (30%)	113 (49%)	< 0.0001	85 (39%)	0.03	0.04
Birth weight	3270 (504) [3315, 2995–3622]	3256 (555) [3325, 2966–3605]	0.76	3276 (572) [3318, 2992–3588]	0.89	0.70
Pre-pregnancy BMI	19.5 (10.4) [22.3, 19.5–25.9]	26.8 (5.2) [26.3, 23.0–30.1]	< 0.0001	27.2 (5.1) [26.9, 23.7–30.1]	< 0.0001	0.45
Country of birth						
Israel	310 (88%)	191 (83%)	0.01	188 (87%)	0.051	0.16
Ethiopia	4 (1.1%)	8 (3.4%)		10 (4.6%)		
USSR	28 (8.0%)	14 (6.0%)		12 (5.6%)		
Other	10 (2.8%)	17 (7.4%)		6 (2.8%)		
Immigrant	42 (12%)	39 (17%)	0.09	28 (13%)	0.72	0.24
Male fetus	181 (52%)	128 (56%)	0.33	110 (53%)	0.89	0.46
Multiple pregnancy	3 (0.9%)	10 (4.4%)	0.005	4 (1.9%)	0.44	0.13
Marital status						
Married	346 (98%)	226 (98%)	0.85	210 (97%)	0.63	0.57
Single	5 (1.4%)	4 (1.7%)		4 (1.9%)		
Divorced	1 (0.3%)	0 (0%)		2 (0.9%)		
Cesarean delivery	41 (12%)	69 (30%)	< 0.0001	62 (29%)	< 0.0001	0.74
GCT mg/dL	100 (26) [96, 81–114]	167 (29) [162, 147–187]	< 0.0001	171 (31) [170, 152–189]	< 0.0001	0.13
OGTT (mg/dL): fasting		88 (13) [85, 79–97]		95 (17) [94, 85–103]		< 0.0001
OGTT: 1-h post glucose load		201 (27) [200, 190–215]		203 (24.5) [202, 189–216]		0.52
OGTT: 2-h post glucose load		157 (31) [159, 135–178]		164 (37) [164, 140–185]		0.06
OGTT: 3-h post glucose load		99 (34) [96, 73–123]		111 (45) [106, 81–131]		0.004
Study outcomes						
Type 2 diabetes mellitus	19 (5.4%)	86 (37%)	< 0.0001	121 (56%)	< 0.0001	< 0.0001
Dyslipidemia	49 (14%)	67 (29%)	< 0.0001	102 (47%)	< 0.0001	< 0.0001
Obesity	111 (32%)	111 (48%)	< 0.0001	124 (57%)	< 0.0001	0.053
Hypertension	44 (13%)	42 (18%)	0.06	39 (18%)	0.07	0.96

Values are presented as mean (standard deviation) [median, IQR] or number (percent)

Missing values: Gender: four missing in control, 2 in good glycemic control, and 7 in poor glycemic control

Mode of delivery: two in good glycemic control, 1 in poor glycemic control

Num. pregnancy: 1 missing in control

Num. birth: 1 missing in control

Pre-pregnancy BMI: 90 missing in control, 15 missing in good glycemic control, 27 missing in poor glycemic control

GCT: In the control group, 15 women had normal GCT according to the medical chart without the exact value, 29 missing in good glycemic control, 43 missing in poor glycemic control

OGTT: Fasting: Twenty-two missing in good glycemic control, 39 missing in poor glycemic control; OGTT: 1-h post glucose load: Sixteen missing in good glycemic control, 36 missing in poor glycemic control

OGTT: 2-h post glucose load: Eighteen missing in good glycemic control, 38 missing in poor glycemic control

OGTT: 3-h post glucose load: Twenty-five missing in good glycemic control, 45 missing in poor glycemic control

* Control vs good glycemic control

^†^Control vs poor glycemic control

^‡^Good glycemic control vs. poor glycemic control

The rates of study outcomes are presented in Table 1. The rate of type 2 diabetes mellitus and dyslipidemia was greater in pregnancies with GDM and, to a greater extent, in pregnancies with poor glycemic control.

**Fig. 3:**
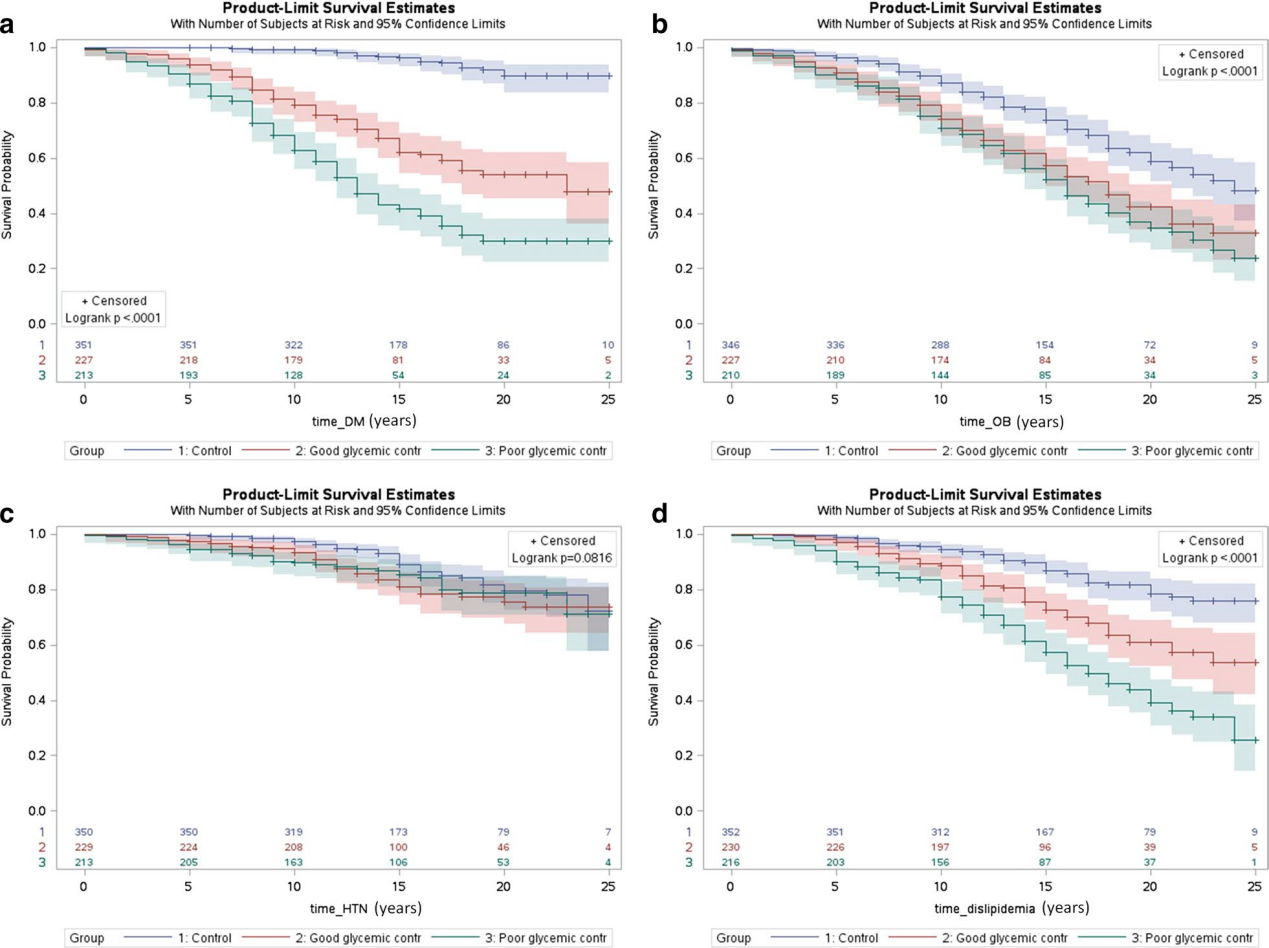
Kaplan Meier survival curve of years from pregnancy until development of type 2 diabetes mellitus (DM) (**a**), obesity (OB) (**b**), hypertension (HTN) (**c**), and dyslipidemia (**d**) for women in the control group, women with GDM with good glycemic control and poor glycemic control. Logrank test for all the comparisons is statistically significant (*p* < 0.05). Control refers to women without gestational diabetes mellitus (GDM) during pregnancy. Excluded from analysis were women with diagnosis prior to the index pregnancy or cases in which the exact date of the diagnosis is unknown

Kaplan–Meier curves for the risks of the control group, GDM with good glycemic control, and GDM with poor glycemic control to develop type 2 diabetes mellitus, obesity, hypertension and dyslipidemia are presented in Fig. 3. The risk of developing all the outcomes apart from hypertension was the greatest for the poor glycemic control group, medium for the good glycemic control group, and the lowest for the control group (log-rank test *p* < 0.05). The Kaplan–Meier curve for the risk to develop ischemic heart disease was not statistically significant between the groups (log-rank test *p* = 0.38).

We explored independent risk factors for the study outcomes by carrying out a stepwise multiple Cox regression on the study group, age, pre-pregnancy BMI, number of previous pregnancies, number of previous births, cesarean delivery, country of birth and emigration. The results are presented in Table 2. GDM pregnancy, glycemic control, pre-pregnancy BMI, and parity were independent risk factors for the development of type 2 diabetes mellitus. Those factors, apart from parity, are also independent risk factors for dyslipidemia. Pre-pregnancy BMI was the only independent risk factor for developing obesity and, alongside maternal age, was also an independent risk factor for hypertension.

**Table 2:** Risk factors during pregnancy for developing characteristics of the metabolic syndrome—multivariate analysis

Risk factor	Adjusted HR [95% CI]	*p* value
Type 2 diabetes mellitus		
Poor glycemic control vs. controls	14.8 [7.6–28.8]	< 0.0001
Good glycemic control vs. controls	8.4 [4.3–16.6]	< 0.0001
Poor glycemic control vs. good glycemic control	1.7 [1.3–2.4]	0.0004
Pre-pregnancy body mass index (BMI)	1.04 [1.01–1.1]	0.004
Parity	1.2 [1.1–1.2]	< 0.0001
Dyslipidemia		
Poor glycemic control vs. controls	3.7 [2.3–5.9]	< 0.0001
Good glycemic control vs. controls	2.0 [1.3–3.3]	0.004
Poor glycemic control vs. good glycemic control	1.8 [1.3–2.6]	0.0003
Pre-pregnancy BMI	1.05 [1.02–1.07]	< 0.0001
Obesity		
Pre-pregnancy BMI	1.09 [1.07–1.1]	< 0.0001
Hypertension		
Pre-pregnancy BMI	1.04 [1.004–1.07]	0.04
Maternal age during pregnancy	1.1 [1.07–1.20]	< 0.0001

Stepwise multiple Cox regression model to evaluate independent risk factors for developing type 2 diabetes mellitus: hypertension, obesity, and dyslipidemia. Controls refer to women without gestational diabetes mellitus during pregnancy. Good glycemic control refers to mean daily glucose charts ≤ 95 mg/dL. Poor glycemic control refers to mean daily glucose charts > 95 mg/dL

*HR* hazard ratio

OGTT values were formerly shown to be indicators for GDM severity [17, 18]. Therefore, we also examined whether glycemic control and OGTT values were independent risk factors for the study outcomes when both are incorporated to the multivariate Cox regression. We found that both glycemic control and the fasting value of the OGTT are independent risk factors for type 2 diabetes mellitus (adjusted HR with 95% CI 1.6 [1.2–2.1] and 1.03 [1.02–1.04], respectively) and dyslipidemia (adjusted HR with 95% CI 1.6 [1.2–2.3] and 1.01 [1.004–1.02], respectively). Finally, we examined the interaction between glycemic control and the OGTT values and did not find it statistically significant (*p* > 0.05 for all the analyses), suggesting that the effect of glycemic control on the development of type 2 diabetes mellitus and dyslipidemia is not affected by the OGTT values.

### Sub-analysis of glycemic control according to both mean daily glucose chart and insulin use

Insulin use for glycemic control represents a poor glycemic control state in and of itself. We therefore performed an additional analysis in which we compared women with good glycemic control without insulin to women with either insulin use or poor glycemic control without insulin. Poor glycemic control according to this definition increased the risk for type 2 diabetes mellitus (HR 2.4 95% CI [1.8–3.3]) and dyslipidemia (HR 1.8 95% CI [1.3–2.5]). The risk for hypertension and obesity was not increased in this group (HR 1.04 95% CI [0.7–1.6] and HR 1.2 95% CI [0.9–1.5], respectively).

### Components of the daily glucose charts and the risk for future metabolic and cardiovascular morbidities

Finally, we examined the association between the mean pre-prandial and post-prandial glucose values of the daily glucose charts and the risk for type 2 diabetes mellitus, dyslipidemia, hypertension and obesity. Of 446 women with mean glucose levels, 328 (74%) had provided glucose reports with information about the pre-prandial and post-prandial glucose levels.

Both mean pre-prandial and post-prandial glucose values were higher in the group with type 2 diabetes mellitus compared to the group without T2DM (89 ± 12 versus 87 ± 11, p = 0.04 and 113 ± 16 versus 107 ± 13, p = 0.0002; respectively).

Mean pre-prandial glucose values were higher in the group with obesity versus the group without obesity (89 ± 11 versus 86 ± 11, p = 0.01; respectively). Mean post-prandial glucose values were higher in the group with dyslipidemia compared to the group without dyslipidemia (113 ± 16 versus 107 ± 14, p = 0.01; respectively).

After adjusting for age, BMI before pregnancy, the number of previous pregnancies, the number of previous births, fasting and 1 h OGTT results and the number of glucose charts for each woman only the association between the mean post-prandial glucose levels and type 2 diabetes mellitus remained significant (adjusted HR with 95% CI 1.015 [1.001–1.029]).

## Discussion

The present study examined the association between the glycemic control of GDM pregnancy and the risk for future metabolic and cardiovascular morbidities. We focused on a novel predictor for metabolic morbidities, the glycemic control according to mean daily glucose in pregnancies with GDM. This variable was not sufficiently studied in the past, probably due to difficulties in collecting data on daily glucose charts which is usually not available retrospectively. The results demonstrated that GDM and poor glycemic control were independent risk factors for the earlier development of type 2 diabetes mellitus and dyslipidemia.

The association between GDM and long-term complications has been described in earlier studies. It was estimated that 50% of patients suffering from GDM will eventually develop type 2 diabetes mellitus [19]. The severity of glycemia in pregnancy as represented by OGTT and GCT values was shown to be positively associated with type 2 diabetes mellitus and with a risk of cardiovascular disease [20–23]. The assessment of ongoing glycemic control as represented by the daily glucose charts adds an additional important, yet less studied insight to this subject. One study reported an increased risk for type 2 diabetes mellitus in women with any 2-h postprandial blood glucose level of 150 mg/dL or higher [24], a result that supports the effect of glycemic control on future type 2 diabetes mellitus and encourages further investigation.

The prevalence of the metabolic syndrome was 2–4 times higher in women with prior GDM after adjusting for possible confounders [7, 8, 25, 26]. Several studies aimed to elucidate possible mechanisms for this observation [27]. Women with GDM demonstrated alterations in cardiometabolic biomarkers; among them, lower levels of serum adiponectin after 1 and 3 years postpartum, rising plasminogen activator inhibitor-1 (PAI-1) over time [28], inflammation markers such as the upregulation of tissue inhibitor of metalloproteinase-1 (TIMP-1) [29] and a higher leptin/adiponectin ratio [30]. A history of GDM did not affect the level of chemerin, retinol-binding protein-4 (RBP-4), C-reactive protein and matrix metalloproteinase-8 (MMP-8) and MMP-9 [28, 29]. changes in haemodynamics amongst pregnant women with GDM such as increased arterial stiffness and blood pressure was also demonstrated [29, 31]. In addition, hypertensive disease in pregnancy was a risk factor for the midlife development of type 2 diabetes mellitus in and of itself [32].

The data was inconsistent with regard to which components of the metabolic syndrome are affected by a history of GDM pregnancy as some studies demonstrated increased risk for all the components [7, 8] while others did not find an effect on dyslipidemia or hyperglycemia [25]. The reasons for this difference might be related to differences in follow-up duration, sample size, GDM diagnostic criteria and the studied population. The current study was designed to address several limitations presented by earlier studies that were concerned with this topic. Firstly, mean follow-up duration was longer than in previous studies. Secondly, the sample size was comparable or greater. Thirdly, most studies addressed the rate of metabolic complications but did not address the duration of time required for their development by calculating hazard ratios. This data is important since it represents the window of opportunity for medical and lifestyle interventions. Since pregnancy is a time of a physiological increase in insulin resistance [33, 34], it serves as a stress test for the body and is a time in which future pathologies might manifest temporarily. Pregnant women are a highly compliant population, which is why pregnancy is an excellent window of opportunity for diagnosis and interventions.

The relation between glycemic control and future complications might be explained by two hypotheses. The first is that women with poor glycemic control have more severe diseases to begin with or are less compliant and are therefore at an increased risk for future complications [20–23]. A second hypothesis is that hyperglycemia during pregnancy is toxic to the pancreas and other tissues due to DNA damage secondary to production of reactive oxygen species [35], which, in turn, leads to the early manifestation of type 2 diabetes mellitus and other complications. The current study cannot distinguish between the two hypotheses and current thinking is that the severity of glycemia in pregnancy reflects an underlying cardiometabolic profile and beta-cell dysfunction. However, it should nonetheless be noted that this is not based on data regarding ongoing glycemic control in pregnancy as such a study was not conducted previously. The observation that glycemic control modified the risk for type 2 diabetes mellitus and dyslipidemia after adjusting for OGTT values, which reflect the fasting glucose and the pancreatic response to a glucose load without being affected by patient compliance or treatment, might point to this direction. A study that examined the effect of social contributors to glycemic control in GDM observed that poor glycemic control was associated with a chaotic lifestyle, the receipt of food stamps, being non married and no regular exercise [36]. Those results, in turn, imply that glycemic control might be associated with modifiable factors and does not simply reflect the severity of the underlying disease and that this hypothesis should be further evaluated in prospective trials. Additional modifiable risk factors for type 2 diabetes mellitus following GDM include body mass, breastfeeding and choice of contraception [37].

The present study set the cutoff value for the development of type 2 diabetes mellitus at 95 mg/dL, which is lower than the acceptable cutoffs for the neonatal short-term outcome [3]. This accords with the findings that even mild forms of hyperglycemia induced measurable DNA damage [35] and unfavorable maternal and neonatal outcomes [38–40]. Indeed, it was suggested that glucose values in pregnant women without GDM are much lower than estimated previously and that target glucose values should be lowered in order to decrease GDM-related complications. Nevertheless, a direct comparison between the current glucose cutoffs and the suggested lower ones still needs to be made [41, 42].

The strengths of this study are the use of longitudinal data from the Gestational Diabetes clinic, which has acted according to a unified protocol in the last 25 years, the availability of glucose charts and information regarding long term outcomes, and the fact that population migration is not common in our area meaning that medical monitoring is conducted at the same hospital and community clinics.

The main limitation of this study is a retrospective design that limits the ability to explore whether strict glycemic control can protect against future metabolic complications, an investigation that would require a randomized controlled trial of women with GDM divided into groups with different target mean glucose values. The feasibility of such a study is questioned as it takes many years for long-term complications to develop. Moreover, the outcomes in this study are based on medical records, which are subject to bias. We examined metabolic and cardiovascular morbidities including type 2 diabetes mellitus, but we did not follow the criteria for metabolic syndrome since it requires information that could not be obtained retrospectively (e.g. documented waist circumference). However, since the follow up for the entire study population took place in the same clinics and was conducted by the same physicians according to the same guidelines and alert systems for abnormal laboratory results, and since Israeli law provides that all citizens are entitled to the same type of national health insurance we believe that this bias is small. The possibility of missing data was addressed by excluding patients without information in the databases. The fact that ongoing surveillance for progression to diabetes is widely recommended for women with a history of GDM is another concern (in our department, it is recommended that every woman with GDM should undergo OGTT at 6–12 weeks postpartum and every year thereafter). Yet, the practice in Israel is that diabetes screening is done regularly to all adults as acknowledged by the Israeli task force for health promotion and preventive medicine [43]. In addition, around 95% of women who were examined in the control group had blood tests that could be analyzed; those without data were excluded. Hence, the difference between the groups in terms of medical availability and accessibility should not be substantial.

## Conclusions

Altogether, this study demonstrated that glycemic control in GDM is an important independent risk factor for future type 2 diabetes mellitus and dyslipidemia. The fact that it is still statistically significant after controlling for the OGTT results, which reflect the baseline disease severity, implies that improving glycemic control might reduce the risk for those outcomes, but this hypothesis should be evaluated in prospective trials.

## Data Availability

The datasets used and analyzed in the course of the present study are available from the corresponding author upon reasonable request.

## Abbreviations

AUC: area under the curve
BMI: body mass index
GCT: glucose challenge test
GDM: gestational diabetes mellitus
HR: hazard ratio
NDDG: National Diabetes Data Group
OGTT: oral glucose tolerance test
ROC: receiver operating characteristic
T2DM: type 2 diabetes mellitus
